# Impact of Matrix Metalloproteinase 9 on COPD Development in Polish Patients: Genetic Polymorphism, Protein Level, and Their Relationship with Lung Function

**DOI:** 10.1155/2018/6417415

**Published:** 2018-12-10

**Authors:** Iwona Gilowska, Łukasz Kasper, Katarzyna Bogacz, Jan Szczegielniak, Teresa Szymasek, Marta Kasper, Marcin Czerwinski, Krzysztof Sładek, Edyta Majorczyk

**Affiliations:** ^1^Institute of Physiotherapy, Faculty of Physical Education and Physiotherapy, Opole University of Technology, Opole, Poland; ^2^Second Department of Internal Medicine, Department of Pulmonology, Collegium Medicum, Jagiellonian University in Cracow Kraków, Poland; ^3^Department of Pulmonology, Opole Voivodship Hospital, Opole, Poland; ^4^Faculty of Health Sciences, Jagiellonian University Medical College, Krakow, Poland; ^5^Ludwik Hirszfeld Institute of Immunology and Experimental Therapy, Polish Academy of Sciences, Wrocław, Poland

## Abstract

Chronic obstructive pulmonary disease (COPD) is characterized by a decline of lung function and symptoms such as chronic bronchitis and emphysema leading from lung tissue destruction. Increased activity of matrix metalloproteinases (MMPs) and an imbalance between MMPs and their tissue inhibitors (TIMPs) are considered as factors influencing the pathogenesis of COPD. We investigated the role of genetic polymorphism and expression level of MMP-9 and concentration of its complexes with TIMPs in the development of COPD among Polish patients. We analyzed SNP in the promoter region of* MMP-9* gene (rs3918242) using PCR-RFLP method among 335 COPD patients and 309 healthy individuals. Additionally, 60 COPD patients and 61 controls were tested for copy number variants (CNV) of* MMP-9* (by quantitative real-time PCR) and serum levels of MMP-9 and its complexes with TIMP1 and TIMP2 (using ELISA). All subjects were analyzed for lung function using spirometry (FEV_1_% and FEV_1_/FVC parameters). We observed that allele and genotype frequencies of the SNP rs3918242, as well as the number of gene copies, were similar in COPD patient and controls groups. Serum levels of MMP-9 and MMP-9/TIMP1 complex were significantly higher in COPD patients in comparison to controls groups, although independently of analyzed gene polymorphisms. Additionally, the significant inverse relationships between parameters of lung function (FEV_1_% and FEV_1_/FVC) and proteins level were found in ridge regression models, especially we found that FEV_1_% decreased when MMP-9 level increased in controls and patients with COPD group. In conclusion, we found that COPD patients were predisposed to produce more MMP-9 and MMP-9/TIMP1 complex than healthy individuals. This phenomenon is probably associated with the disease-related lung environment but not with genetic features of the* MMP-9*.

## 1. Introduction

Chronic obstructive pulmonary disease (COPD) is characterized by airflow limitation through the respiratory tract that is not fully reversible. The disease is associated with lung inflammatory response to harmful gases and dust, including cigarette smoke [1]. Moreover, COPD patients are affected with a pulmonary emphysema leading from lung tissue destruction and the decline of lung function as a result [2, 3]. Thus, it is generally believed that mechanism underlying the COPD pathogenesis is dysregulation of proteases activity, especially when imbalance between proteases and antiproteases develops because of either the higher activity of proteases or dysfunction of protease inhibitors [3–5].

It is widely accepted that metalloproteinases (MMPs) engaged in the COPD pathogenesis degrade matrix proteins (elastin, collagen) and the metalloproteinase 9 (MMP-9) plays a principal role in this process [6]. The protease is a type IV collagen-degrading enzyme, and its presence is required for adequate matrix processing and lung repair [7, 8]. However, MMP-9 oversecretion may cause an unwanted degradation of lung tissue, and this may be one of the causes of COPD [6], probably due to the tobacco smoke stimuli [4]. The increased expression of MMP-9 may be associated with an increasing level of gene promoter activity. In particular, the -1562C/T polymorphism (rs3918242) in the* MMP-9* gene promoter was found to be associated with MMP-9 expression, and the -1562T allele leads to higher transcription activity [9].

In this study, we evaluated the role of* MMP-9* gene -1562C/T polymorphism, as well as MMP-9 protein and its complexes with TIMP levels, in COPD development in Polish patients.

## 2. Materials and Methods

### 2.1. COPD Patient and Controls Group

Three hundred thirty-five patients (248 males and 87 females) with COPD were enrolled in the study. All subjects underwent routine diagnosis including the spirometry result and FEV_1_/FVC ratio reduction below the lower limit of the norm. The spirometry test was performed twice, before the bronchodilator application (400 *μ*g of salbutamol) and after (15 – 20 minutes). The outcome of the bronchial relaxation trial performed in that way should be negative. Patients with alpha-1- antitrypsin deficiency, coexistence of asthma, and COPD or with earlier history of bronchial asthma as well as patients who have never smoked were excluded from the study. All patients were recruited from the inpatient and outpatient populations of the University Hospital in Cracow, Specialized Hospital of Ministry of Internal Affairs and Administration (MSWiA) in Głuchołazy and Department of Pulmonology, Opole Voivodeship Hospital.

Three hundred and nine unrelated healthy volunteers (80 men and 229 women) not diagnosed for COPD nor any other lung disease and exhibiting normal lung function (mean FEV_1_% of 91.7%) served as a control group. To determine the effect of an exposure to tobacco smoke on analyzed factors, the controls were divided into subgroups of those with tobacco smoking history (110 current and former smokers) and those who had never smoked (199 never smokers).

For CNV and protein levels examination, 60 patients with COPD and 61 control individuals (23 smokers and 38 never smokers) were randomly selected.

All members of the patients and control group were of Polish Caucasians ethnicity, and detailed characteristics are shown in Table 1.

The project was approved by the Ethics Committee of Opole Voivodship. Signed informed consent was obtained from all persons tested.

### 2.2. DNA Isolation and SNP and CNV Genotyping

gDNA was extracted from whole blood using the GeneMATRIX Quick Blood DNA Purification Kit (EURx, Poland) following the manufacturer's instructions. The -1562C/T polymorphism of the* MMP-9* gene (rs3918242) was typed by the PCR-RFLP method as described previously [9]. Briefly, polymerase chain reactions were carried out in 20 *μ*l of solution containing a standard PCR buffer, 200 ng of tested DNA, 1 U of Taq Polymerase (Invitrogen, Brazil), 2 mM MgCl_2_, 200 *μ*M dNTPs (Invitrogen, Brazil), and 1 *μ*M of each primer (forward: 5′-GCCTGGCACATAGTAGGCCC-3′ and reverse: 5′-CTTCCTAGCCAGCCGGCATC-3′). Amplifications were performed under the following conditions: 2 min at 94°C; 30 cycles of 30 s at 94°C; 30 s at 60°C; 60 s at 72°C with the last elongation step of 7 min at 72°C. The PCR product was digested with 1 U of SphI restriction enzyme (Fermentas, USA) for 3 h at 37°C (435 bp amplicons containing a T allele were cut into two fragments of 244 bp and 191 bp). All digested products were electrophoresed on 3% agarose gel and visualized in a UV transilluminator using ethidium bromide staining.

The CNV polymorphism was typed using the quantitative polymerase chain reaction (qPCR) by TaqMan Copy Number Assays (Applied Biosystems of Life Technology, Foster City, CA) according to the manufacturer's protocol in total reaction volume of 20 *μ*L (contained 20 ng gDNA, 1x probes for both the target (hs 00238040cn) and reference gene (RNase P) and 1x TaqMan Genotyping Master Mix). The PCR program was processed as follows: 95°C for 10 min, 40 cycles of 95°C for 15 s, and 60°C for 1 min. Data were collected at the end of each cycle. All DNA samples were run in triplicate to ensure accurate results. Then, the CopyCaller Software (Applied Biosystems by Life Technology, CA) was used to analyze copy numbers of the tested gene in the samples.

### 2.3. Analysis of Protein Levels

The serum level of MMP-9 was measured using the Quantikine enzyme-linked immunosorbent assay (ELISA), and serum concentrations of its complexes with TIMP1 and TIMP2 were determined by DuoSet ELISAs (all R&D, MN, USA). All analyses were performed according to the manufacturer's instruction using serum samples either nondiluted (for MMP-9) or diluted of 100-fold (for complexes). The absorbance was read with the Epoch microplate reader (BioTech, Winooski, VT, USA) at 450 nm as the primary wave length and 540 nm as the reference. Concentrations of proteins in samples were calculated with the equation of the standard curve using Gen5 software (BioTech, Winooski, VT, USA).

### 2.4. Statistical Analysis

Deviation of the genotype counts from Hardy-Weinberg equilibrium was tested using the *χ*^2^- test and EpiInfo software. Differences in alleles and genotypes distribution between controls and patient groups were estimated using the two-tailed Fisher's exact test and *χ*^2^-test with Yate's correction, respectively. GraphPad InStat 3 software was used for distribution analyses. Moreover, the multiple inheritance models (codominant, dominant, recessive, overdominant, and log-additive) via logistic regression with binary response (a disease status) using SNPstats web tool (https://www.snpstats.net) were estimated. In protein levels, before relevant statistical comparison of all obtained data, the distribution of values was checked with the method of Shapiro-Wilk. One-way analysis of variance (ANOVA) was used to assess differences between obtained values of protein concentration. Subsequently, when significant interactions in ANOVA were identified, appropriate Tukey post hoc tests were applied. For the analysis of relationship between protein levels and lung function parameters, a multiple ridge regression model was used. For analysis of COPD development predictors, a logistic regression fitted with the disease status as a response variable was used and as covariates were selected: SNP rs3918242, CNV, gender, smoking status and levels of MMP-9, and its complexes with TIMP1 and TIMP2. These statistical analyses were performed using Statistica v.12 (StatSoft, Inc., OK, USA). In all analyses, p values ≤0.05 were considered significant.

## 3. Results

In total, 335 patients affected with COPD and 309 healthy individuals were typed for SNP rs3918242 in the promoter of the gene encoding MMP-9. The observed genotype frequencies were very close to the values expected for both cases and controls populations according to the Hardy-Weinberg equilibrium (p<0.05, data not shown). There were no significant differences in alleles and genotypes frequency distribution between the COPD patients and controls (Table 2). When controls were divided into two groups according to the smoking status, nonsignificantly lower -1562C allele frequency in smokers was observed (83.2% versus 87.1% in COPD group,* p = 0.09*; Table 2). Additionally, no differences between patients with COPD and controls in genotype frequencies distribution were observed in dominant (CC versus CT+TT), recessive (CC+CT versus TT), and overdominant (CC+TT versus CT) models of inheritance (Table 1S in Supplementary Material).

The numbers of* MMP-9* gene copies were analyzed in the groups of 60 randomly selected patients with COPD and 61 healthy volunteers. We found that 85.0% of COPD patients and 82.0% of controls had 2 copies of the* MMP-9* gene. Additionally, we also found individuals with 1 copy (3.3% and 4.9% in patients and controls, respectively), 3 copies (11.7% and 9.9% in patients and controls, respectively), and 4 copies (3.2% of controls). However, no significant difference in CNV frequency between COPD patients and the control group was found (Table 2).

We also evaluated the levels of MMP-9 and its complexes with TIMP1 and TIMP2 in serum of COPD patients and controls (the same as selected for CNV) (Table 3). We found that the mean serum MMP-9 levels in the COPD group were significantly higher in comparison with the control group (149.0 ng/ml versus 26.5 ng/ml; p < 0.0001), as well as those of the controls subgroups with smoking status (27.5 ng/ml in smokers and 25.9 ng/ml in never smokers, p = 0.37 for comparison between both control subgroups). In contrast, there were no significant differences in the mean serum levels of MMP-9/TIMP1 and MMP-9/TIMP2 between the COPD patients and controls, except a significant difference between COPD patients and total controls in levels of MMP-9/TIMP1 complex (3146.8 pg/ml versus 2970.1 pg/ml, p = 0.04). Additionally, serum of control smokers contained a significantly higher level of this complex in comparison to control nonsmokers (3135.8 pg versus 2869.8 pg, respectively; p = 0.03; Table 3).

In particular, in systematic comparison between SNP rs3918242, CNV genotypes, and serum MMP-9, MMP-9/TIMP1 and MMP-9/TIMP2 levels showed no statistically significant differences (Table 4). However, the COPD patients with 2 copies of the* MMP-9* gene exhibited lower MMP-9 serum level in comparison to the combined group of patients with 1 or more than 2 copies of the gene (142.9 ng/ml versus 186.8 ng/ml, p = 0.09; Table 4).

Generally, COPD patients were characterized by significantly lower lung function parameters (FEV_1_% and FEV_1_/FVC) in comparison to controls (Table 1). This phenomenon continued to be observed after division of groups according to SNP rs3918242 and CNV genotypes. On the other hand,* MMP-9* genotypes-related intragroup comparisons did not reveal any significant differences (Table 4).

In addition, interactions between lung function parameters and levels of MMP-9, MMP-9/TIMP1, and MMP-9/TIMP2 were analyzed using ridge regression modeling. In controls group, an increment in MMP-9 levels was significantly associated with lower FEV_1_% (when MMP-9 level increases by 1 unit, the FEV_1_% decreases by 0.86). The increase of levels of complexes MMP-9/TIMP1 and MMP-9/TIMP2 was negatively related to FEV_1_/FVC parameter, which decreases by 0.01 and 2.50 with each additional unit of MMP-9/TIMP1 and MMP-9/TIMP2, respectively (Table 5). In patients with COPD group, the model showed a significant negative relationship between MMP-9 levels and values of both lung function parameters (FEV_1_% decreases by 0.06 and FEV_1_/FVC decreases by 0.04 for increased by 1-unit protein level). Additionally, MMP-9/TIMP1 complex level was positively related to FEV_1_/FVC, which increases by 0.006 for every 1-unit increasing in the complex level (Table 5).

Table 6 shows a logistic regression analysis of potential predictors of COPD development; the variables found to be significantly associated with the COPD in univariate logistic regression analysis were gender (OR = 8.16, 95% CI = 5.73-11.61, p < 0.0001), smoking (OR = 606.04, 95% CI = 83.96-4374.76, p < 0.0001), and MMP-9 protein level (OR = 1.10, 95% CI = 1.06-1.14, p < 0.0001). Taking these covariates with significant association in univariable analysis into multivariable analysis revealed that MMP-9 serum level was considered as a potential independent risk factor (OR = 1.12, 95% CI = 1.03-1.23, and p = 0.001).

## 4. Discussion

It is postulated that the action of MMPs can lead to tissue degradation and emphysema and provide physiological remodeling of lung tissue, but when misregulated, may be associated with COPD development and severity. The protease-antiprotease imbalance is at the focus point of COPD pathogenesis hypothesis [10]. A classic example of this phenomenon is deficiency of the serine protease inhibitor, *α*1-antitrypsin, which is commonly known as a major genetic factor in emphysema development [11] but accounts for only 3% of COPD patients. Thus, it seems that the crucial role in the pathogenesis of COPD is played by other proteases and their specific inhibitors. The role played by MMP-9 and its inhibitors TIMP1 and TIMP2 seems to be particularly important [9, 11]. MMP-9 is an enzyme secreted by several cell types, including neutrophils and airway epithelial cells, as well as macrophages, which can be involved in COPD pathogenesis. In an attempt to resolve that issue, we performed analysis of genetic and physiological features of MMP-9 in COPD pathogenesis.

In genetic analysis, our results suggest that there is no association between alleles and genotypes of the SNP rs3918242 (-1562 C/T) and COPD susceptibility among Polish COPD patients. This finding is in accordance with other studies on the Caucasian population with the exception of non-Hispanic Whites and Hispanic veterans, in case of whom it was concluded that TT genotypes constitute a risk factor for COPD development [12]. In contrast, the -1562T allele seems to be associated with the disease in Asians, but with some ambiguity in Koreans the T allele seems to play a protective role (reviewed by Stankovic et al. [13]). Nevertheless, two meta-analyses showed that SNP rs3918242 is associated with the risk of COPD [14, 15].

Despite this contradictory role of mentioned polymorphism [6, 12, 14–17], this SNP is most widely analyzed SNP of* MMP-9* gene and it is considered as the functional promoter polymorphism. When T allele is present, the higher promoter activity has been shown [9, 13], and it also was shown that COPD patients with the CT genotype expressed a higher serum level of MMP-9 [18]. Our results did not support this finding: we confirmed the results obtained by other authors [19–22] that the MMP-9 level in serum of COPD patients is higher in serum than in healthy controls, but we found that the difference does not depend on the SNP rs3918242, as well as on the number of* MMP*-9 gene copies (Table 4). Thus, we hypothesize that the increased secretion of MMP-9 in COPD patients may be a result of the disease-specific lung microenvironment, and this phenomenon could be explained by an inducible character of this enzyme [4].

The MMP is not produced by resident pulmonary macrophages in healthy lung; in contrast, in the COPD-related lung microenvironment, immunocompetent cells are the main source of MMP-9 [23]. It is known that stimulation of neutrophils leads to release of MMP-9 [24] and the alveolar macrophages from COPD patients produce more MMP-9 than macrophages from healthy controls in response to either inflammatory stimuli or cigarette smoke [25]. Therefore, it is suggested that MMP-9 serum level is associated with the disease severity and it is likely that it negatively correlates with lung function parameters (FEV_1_%, FEV_1_/FVC) [20, 26, 27]. In a compliance with this indication, we found the inverse relationship between MMP-9 level and FEV_1_% in both groups (patients with COPD and controls) and additionally with FEV_1_/FVC in COPD patients. Because of this, it cannot be excluded that MMP-9 is a biomarker for the presence and clinical course of COPD. Moreover, our result suggests that the enzyme level possesses a capability to be an independent predictor of the disease development. In aspect of this finding, it should be kept in mind that COPD is particularly associated with smoking (the variable included to our regression analysis). It is known that polymorphonuclear leukocytes from healthy controls could be stimulated by cigarette smoke to secrete MMP-9 at a level similar to the one found in COPD patients [4], but we did not find the different concentrations of MMP-9 in smokers and nonsmokers subgroups of controls. Nevertheless, it cannot be excluded that elevated level of MMP-9 accompanied smoking. Therefore, our regression observations need to be replicated, in the same or other populations, in order to consider MMP-9 as the biomarker and/or the predictor of COPD to reflect the complex role of MMP-9 in COPD.

MMP-9 is inhibited by the tissue inhibitors of metalloproteinase (TIMP1) and so the imbalance between MMP-9 and TIMP1 ratio could be involved in COPD pathogenesis [28]. Herein, in complement of the MMP-9 and TIMP1 ratio analysis [28, 29], we demonstrate that the concentration of the MMP-9/TIMP1 complex in the serum of COPD patients is higher than that in healthy controls, which corroborates the findings of other authors [30]. More complexes of MMP-9/TIMP1 could be delivered by increased secretion of MMP-9, but it was also found that COPD patients exhibited a significantly higher level of unbound TIMP1 ([29, 30] and our unpublished data). Additionally, Lo et al. identified that smokers who exhibit the airway hyperresponsiveness produce increased level of MMP-9 to TIMP1, and the authors concluded that this finding indicates a new predictor to identify smokers vulnerable to COPD [31]. Interestingly, our results suggest that the MMP-9/TIMP1 complex concentration seems to be dependent on cigarette smoking (controls smokers exhibit a significantly higher level than nonsmokers).

Several limitations of the present study should be taken into account. A potential important weakness is the design of the study performed for protein levels analysis. We measured MMP-9 and its complexes with TIMP1 and TIMP2 only at the protein level in blood, but not* in situ *using either sputum or bronchoalveolar lavage, which should better reflect the phenomena occurring in the lungs. Nevertheless, no significant difference between serum and induced sputum concentration of MMP-9 was found in a previous study [32]. In addition, despite the fact that in our study groups gender mismatching occurred, no intergender difference in analyzed factors was observed (Figures 1S-5S in Supplementary Material), which is in accordance with the study by de Torres et al. [33], who found similar level of MMP-9 in women and men. Nevertheless, it seems that COPD phenotype depends on sex and men have milder dyspnea and better quality of life than women with a similar degree of airflow obstruction [33]. On the other hand, as we showed male gender could be considered as potential risk factor for COPD development.

## 5. Conclusions

We found that patients affected with COPD were predisposed to produce more MMP-9 and MMP-9/TIMP1 complex than healthy individuals. This phenomenon is probably associated with the disease-related lung environment, but not with genetic features of* MMP-9* gene. Moreover, to the best of our knowledge, this is the first study in which copy number variation of the* MMP-9* gene in the aspect of COPD development was investigated.

## Figures and Tables

**Table 1:** Characteristics of the analyzed groups.

Groups (N)	Age	Sex	FEV_1_	FEV_1_/FVC	Smokers
	(years)	(women/men)	(% predicted)	(% predicted)	(%)
COPD (335)	67.9 ± 9.2	248/87*∗*	55.5 ± 18.5*∗*	52.2 ± 16.0*∗*	100*∗*
CTR (309)	68.0 ± 6.6	80/229	91.7 ± 22.8	89.2 ± 25.8	35.5
CTR smokers (110)	68.4 ± 5.2	73/37	93.3 ± 22.0	91.1 ± 19.5	100
CTR non-smokers (199)	65.8 ± 9.6	157/42	91.0 ± 23.6	88.1 ± 28.7	0
COPD^#^ (60)	70.4 ± 9.0	13/47*∗*	46.6 ± 18.5*∗*	58.1 ± 16.4*∗*	100*∗*
CTR^#^ (61)	67.8 ± 6.7	46/15	90.4 ± 22.5	92.6 ± 23.2	37.7
CTR smokers^#^ (23)	67.7 ± 5.6	15/8	93.2 ± 21.9	88.2 ± 16.4	100
CTR non-smokers^#^ (38)	67.9 ± 7.6	31/7	90.6 ± 22.0	90.7 ± 18.0	0

N, numbers of individuals; COPD, chronic obstructive pulmonary disease groups; CTR, control group; ^#^, groups for CNV and protein levels analyses; FEV_1_, forced expiratory volume in 1 second; FEV_1_/FVC, Tiffeneau-Pinelli index; ratio of forced expiratory volume in 1 second and forced vital capacity; *∗*, difference to the control group statistically significant at the level of p<0.00001.

**Table 2:** Alleles and genotypes distributions of -1562C/T SNP of *MMP-9* gene (rs3918242) and copy number variability of *MMP-9* gene in COPD patient and healthy control groups.

*MMP-9* gene polymorphisms	Frequency (Number of positive)				p value			
	COPD N=335/60^#^	CTR N=309/61^#^	CTR smokers N=110/23^#^	CTR non-smokers N=199/38^#^	p_1_	p_2_	p_3_	p_4_
**rs3918242 (-1562 C/T)**								
**Alleles**								
C	87.1 (583)	85.2 (526)	83.2 (183)	86.1 (343)	0.36	0.09	0.76	0.34
T	12.9 (87)	14.8 (92)	16.8 (37)	13.8 (55)				
**Genotypes**								
CC	76.2 (255)	73.2 (226)	69.1 (76)	75.3 (150)	0.61	0.34	0.36	0.43
CT	21.8 (73)	23.9 (74)	28.2 (31)	21.6 (43)				
TT	2.0 (7)	2.9 (9)	2.7 (3)	10.9 (6)				
**CNV** ^#^								
1 copy	3.3 (2)	4.9 (3)	0.0 (0)	7.9 (3)	0.51	0.31	0.41	0.31
2 copies	85.0 (51)	82.0 (50)	91.3 (21)	76.3 (29)				
3 copies	11.7 (7)	9.9 (6)	4.3 (1)	13.2 (5)				
4 copies	0 (0)	3.2 (2)	4.3 (1)	2.6 (1)				

N, number of individuals; COPD, chronic obstructive pulmonary disease groups; CTR, control group; *p*_1_, COPD patients versus CTR; *p*_2_, COPD patients versus CTR smokers; *p*_3_, COPD patients versus CTR nonsmokers; *p*_4_, CTR smokers versus CTR non-smokers; ^#^, CNV analyses were performed on 60 COPD patients and 61 controls (23 smokers and 38 nonsmokers).

**Table 3:** MMP-9, MMP-9/TIMP1, and MMP-9/TIMP2 proteins level in serum of COPD patient and healthy control groups.

Proteins levels	Groups				p value			
	COPD patients N=60	CTR N=61	CTR smokers N=23	CTR non-smokers N=38	p_1_	p_2_	p_3_	p_4_
**MMP-9 **[ng/ml]	149.0 ± 72.3	26.5 ± 6.5	27.5 ± 7.9	25.9 ± 5.6	<0.00001	<0.00001	<0.00001	0.37
**MMP-9/TIMP1 **[pg/ml]	3146.8 ± 491.9	2970.1 ± 485.1	3135.8 ± 180.4	2869.8 ± 578.8	0.04	0.9	0.10	0.03
**MMP-9/TIMP2 **[ng/ml]	6.1 ± 4.7	6.1 ± 2.0	5.8 ± 1.4	6.3 ± 2.3	0.9	0.6	0.82	0.31

N, number of individuals; COPD, chronic obstructive pulmonary disease groups; CTR, control group; *p*_1_, COPD patients versus CTR; *p*_2_, COPD patients versus CTR smokers; *p*_3_, COPD patients versus CTR nonsmokers; *p*_4_, CTR smokers versus CTR nonsmokers.

**Table 4:** MMP-9 gene polymorphisms impact on MMP-9, MMP-9/TIMP1 and MMP-9/TIMP2 proteins level in serum of COPD patient and healthy control groups.

Proteins levels Genotypes	Groups				p value			
	COPD patients N=60	CTR N=61	CTR smokers N=23	CTR non-smokers N=38	p_1_	p_2_	p_3_	p_4_
**MMP-9 **[ng/ml]								
C allele	149.9 ± 72.8	26.5 ± 6.8	27.6 ± 8.3	25.9 ± 5.8	<0.00001	<0.00001	<0.00001	1.0
T allele	130.7 ± 35.9	27.6 ± 8.3	30.4 ± 8.3	22.6 ± 6.3	<0.00001	<0.00001	<0.00001	0.76
CC	152.9 ± 76.9	26.0 ± 5.6	23.9 ± 5.9	26.7 ± 5.4	<0.00001	<0.00001	<0.00001	1.0
CT	130.7 ± 35.9	27.7 ± 9.3	31.0 ± 8.9	20.5 ± 5.9	<0.00001	<0.00001	<0.00001	1.0
TT	-	27.4 ± 2.5	26.9 ± 1.8	27.9 ± 3.9	not tested	not tested	not tested	0.72
2 copies	142.9 ± 64.9	27.1 ± 6.5	27.5 ± 8.3	26.8 ± 5.0	<0.00001	<0.00001	<0.00001	1.0
1 and >2 copies	186.8 ± 101.8	24.2 ± 6.8	27.6 ± 1.7	23.5 ± 7.4	<0.00001	0.006	<0.00001	1.0
**MMP-9/TIMP1 **[pg/ml]								
C allele	3146.8 ± 492.0	2951.4 ± 496.1	3125.0 ± 182.5	2850.1 ± 588.3	0.14	1.0	0.02	0.18
T allele	3232.3 ± 354.0	3072.8 ± 523.1	3181.7 ± 173.3	2870.6 ± 856.2	not tested (ANOVA p>0.05)			
CC	3131.7 ± 513.8	2920.0 ± 464.0	3076.2 ± 180.3	2869.7 ± 516.3	not tested (ANOVA p>0.05)			
CT	3232.3 ± 354.0	3031.8 ± 578.9	3169.6 ± 181.1	2728.9 ± 1003.9	not tested (ANOVA p>0.05)			
TT	-	3236.7 ± 111.6	3248.4 ± 147.3	3229.0 ± 122.9	not tested (ANOVA p>0.05)			
2 copies	3132.9 ± 532.7	2968.6 ± 512.7	3129.6 ± 187.2	2852.0 ± 633.6	not tested (ANOVA p>0.05)			
1 and >2 copies	3225.9 ± 54.5	2976.9 ± 352.6	3200.6 ± 74.2	2927.3 ± 373.4	not tested (ANOVA p>0.05)			
**MMP-9/TIMP2 **[ng/ml]								
C allele	6.2 ± 4.2	6.2 ± 2.0	5.8 ± 1.4	6.5 ± 2.3	not tested (ANOVA p>0.05)			
T allele	9.4 ± 8.2	5.7 ± 1.8	5.9 ± 1.3	5.5 ± 2.6	not tested (ANOVA p>0.05)			
CC	5.6 ± 2.8	6.3 ± 2.2	5.6 ± 1.6	6.5 ± 2.3	not tested (ANOVA p>0.05)			
CT	9.4 ± 8.2	6.0 ± 1.8	6.0 ± 1.3	6.2 ± 2.8	not tested (ANOVA p>0.05)			
TT	-	4.5 ± 1.2	5.1 ± 0.2	3.9 ± 1.7	not tested (ANOVA p>0.05)			
2 copies	6.3 ± 4.5	6.0 ± 1.6	5.8 ± 1.4	6.0 ± 1.7	not tested (ANOVA p>0.05)			
1 and >2 copies	5.5 ± 0.4	6.8 ± 3.5	5.0 ± 0.5	7.1 ± 3.8	not tested (ANOVA p>0.05)			

N, number of individuals; COPD, chronic obstructive pulmonary disease groups; CTR, control group; *p*_1_, COPD patients versus CTR; *p*_2_, COPD patients versus CTR smokers; *p*_3_, COPD patients versus CTR nonsmokers; *p*_4_, CTR smokers versus CTR nonsmokers.

**Table 5:** Relationships between FEV_1_% and FEV_1_/FVC scores and proteins levels in COPD patient and controls groups.

	Parameters relationship		Protein concentrations					
			COPD patients			Controls		
			MMP-9 [ng/ml]	MMP-9/TIMP1 [pg/ml]	MMP-9/TIMP2 [ng/ml]	MMP-9 [ng/ml]	MMP-9/TIMP1 [pg/ml]	MMP-9/TIMP2 [ng/ml]
Lung function	FEV_1_ [% predicted]	*β*	**-0.06** ^**1**^	-0.01	0.01	**-0.86** ^**3**^	0.01	-0.03
		p	**0.04**	0.30	0.35	**0.04**	0.24	0.07
	FEV_1_/FVC [% predicted]	*β*	**-0.04** ^**2**^	**0.006** ^**2**^	0.005	0.02	**-0.01** ^**4**^	**-2.50** ^**4**^
		p	**0.04**	**0.04**	0.72	0.38	**0.04**	**0.04**

COPD, chronic obstructive pulmonary disease groups; FEV_1_, forced expiratory volume in 1 second; FEV_1_/FVC, Tiffeneau-Pinelli index – ratio of forced expiratory volume in 1 second and forced vital capacity; *β*, coefficients of the regression; ^1^, R^2^ = 0.91, estimated FEV_1_% = 62.20 - 0.06*∗*MMP-9; ^2^, R^2^ = 0.92, estimated FEV_1_/FVC = 44.65 + 0.0006*∗*MMP-9/TIMP1 - 0.04*∗*MMP-9; ^3^, R^2^ = 0.89, estimated FEV_1_% = 113,42 -0.86*∗*MMP-9; ^4^, R^2^ = 0.91, estimated FEV_1_/FVC = 81.13 - 0.01*∗*MMP-9/TIMP1 - 2.50*∗*MMP-9/TIMP2; significant data were bolded.

**Table 6:** Logistic regression analysis for prediction of COPD.

Predictor	Univariable			Multivariable		
	*P*	OR	95% CI	*P*	OR	95% CI
Gender	<0.0001	8.16	5.73-11.61	0.72	1.22	0.06-26.24
Smoking status	<0.0001	606.04	83.96-4374.76	1.0	-	-
MMP-9 level	<0.0001	1.10	1.06-1.14	0.001	1.12	1.03-1.23
MMP-9/TIMP1 level	0.06	1.00	1.00-1.00			
MMP-9/TIMP2 level	0.93	1.00	0.90-1.12			
SNP rs3918242	0.34	0.16	0.85-1.59			
CNV	0.58	0.81	0.38-1.72			

N, number of individuals; COPD, chronic obstructive pulmonary disease groups; CTR, control group; MMP-9, metalloproteinase 9; MMP-9/TIMP1, complex of metalloproteinase 9 and tissue inhibitor of metalloproteinase 1; MMP-9/TIMP2, complex of metalloproteinase 9 and tissue inhibitor of metalloproteinase 2; CNV, copy number variation.

## Data Availability

Data are available in Institute of Physiotherapy, Faculty of Physical Education and Physiotherapy, Opole University of Technology, Proszkowska 76 PL-45-758 Opole, Poland.

## Supplementary Materials

Supplementary Materials Table 1S. Logistic regression analysis of association between 1562C/T SNP of MMP9 gene (rs3918242) and COPD, the multiple inheritance models. Figure 1S. Gender-related frequency of alleles of MMP9 rs3918242 polymorphism (-1562C/T) in patients with COPD and controls groups Figure 2S. Gender-related frequency of genotypes of MMP9 rs3918242 polymorphism (-1562C/T) in patients with COPD and controls groups. Figure 3S. Gender-related MMP-9 protein levels in patients with COPD and controls groups. Figure 4S. Gender-related MMP-9/TIMP1 complex levels in patients with COPD and controls groups. Figure 5S. Gender-related MMP-9/TIMP2 complex levels in patients with COPD and controls groups.

## References

[1] Vestbo J., Hurd S. S., Agustí A. G. (2013). Global strategy for the diagnosis, management, and prevention of chronic obstructive pulmonary disease: GOLD executive summary. *American Journal of Respiratory and Critical Care Medicine*.

[2] Rhee C. K., Kim K., Yoon H. K. (2017). Natural course of early COPD. *International Journal of Chronic Obstructive Pulmonary Disease*.

[3] Navratilova Z., Kolek V., Petrek M. (2016). Matrix Metalloproteinases and Their Inhibitors in Chronic Obstructive Pulmonary Disease. *Archivum Immunologiae et Therapia Experimentalis*.

[4] Somborac-Bačura A., Popović-Grle S., Zovko V., Žanić-Grubišić T. (2018). Cigarette Smoke Induces Activation of Polymorphonuclear Leukocytes. *Lung*.

[5] Pandey K. C., De S., Mishra P. K. (2017). Role of Proteases in Chronic Obstructive Pulmonary Disease. *Frontiers in Pharmacology*.

[6] Kukkonen M. K., Tiili E., Vehmas T., Oksa P., Piirilä P., Hirvonen A. (2013). Association of genes of protease-antiprotease balance pathway to lung function and emphysema subtypes. *BMC Pulmonary Medicine*.

[7] Ishii T., Abboud R. T., Wallace A. M. (2014). Alveolar macrophage proteinase/antiproteinase expression in lung function and emphysema. *European Respiratory Journal*.

[8] Blázquez-Prieto J., López-Alonso I., Amado-Rodríguez L. (2018). Impaired lung repair during neutropenia can be reverted by matrix metalloproteinase-9. *Thorax*.

[9] Zhang B., Ye S., Herrmann S.-M. (1999). Functional polymorphism in the regulatory region of gelatinase B gene in relation to severity of coronary atherosclerosis. *Circulation*.

[10] Fischer B. M., Pavlisko E., Voynow J. A. (2011). Pathogenic triad in COPD: oxidative stress, protease-antiprotease imbalance, and inflammation. *International Journal of Chronic Obstructive Pulmonary Disease*.

[11] Hatipoğlu U., Stoller J. K. (2016). *α*1-antitrypsin deficiency. *Clinics in Chest Medicine*.

[12] Tesfaigzi Y., Myers O. B., Stidley C. A. (2006). Genotypes in matrix metalloproteinase 9 are a risk factor for COPD.. *International Journal of Chronic Obstructive Pulmonary Disease*.

[13] Stankovic M., Kojic S., Djordjevic V. (2016). Gene-environment interaction between the MMP9 C–1562T promoter variant and cigarette smoke in the pathogenesis of chronic obstructive pulmonary disease. *Environmental and Molecular Mutagenesis*.

[14] Chen L., Wang T., Liu L., Shen Y., Wan C., Wen F. (2013). Matrix metalloproteinase-9 -1562C/T promoter polymorphism confers risk for COPD: a meta-analysis. *PLoS ONE*.

[15] Zhou H., Wu Y., Jin Y. (2013). Genetic Polymorphism of Matrix Metalloproteinase Family and Chronic Obstructive Pulmonary Disease Susceptibility: a Meta-analysis. *Scientific Reports*.

[16] Lee S.-Y., Kim M.-J., Kang H.-G. (2010). Polymorphisms in matrix metalloproteinase-1, -9 and -12 genes and the risk of chronic obstructive pulmonary disease in a Korean population. *Respiration*.

[17] Ito I., Nagai S., Handa T. (2005). Matrix metalloproteinase-9 promoter polymorphism associated with upper lung dominant emphysema. *American Journal of Respiratory and Critical Care Medicine*.

[18] Bchir S., Nasr H. B., Hakim I. R. (2015). Matrix Metalloproteinase-9 (279R/Q) Polymorphism is Associated with Clinical Severity and Airflow Limitation in Tunisian Patients with Chronic Obstructive Pulmonary Disease. *Molecular Diagnosis & Therapy*.

[19] D'Armiento J. M., Goldklang M. P., Hardigan A. A. (2013). Increased Matrix Metalloproteinase (MMPs) levels do not predict disease severity or progression in emphysema. *PLoS ONE*.

[20] Koo H., Hong Y., Lim M. N., Yim J., Kim W. J. Relationship between plasma matrix metalloproteinase levels, pulmonary function, bronchodilator response, and emphysema severity. *International Journal of Chronic Obstructive Pulmonary Disease*.

[21] Hernández-Montoya J., Pérez-Ramos J., Montaño M. (2015). Genetic polymorphisms of matrix metalloproteinases and protein levels in chronic obstructive pulmonary disease in a Mexican population. *Biomarkers in Medicine*.

[22] Brajer B., Batura-Gabryel H., Nowicka A., Kuznar-Kaminska B., Szczepanik A. (2008). Concentration of matrix metalloproteinase-9 in serum of patients with chronic obstructive pulmonary disease and a degree of airway obstruction and disease progression. *Journal of Physiology and Pharmacology*.

[23] Atkinson J. J., Lutey B. A., Suzuki Y. (2011). The role of matrix metalloproteinase-9 in cigarette smoke-induced emphysema. *American Journal of Respiratory and Critical Care Medicine*.

[24] Van Den Steen P. E., Dubois B., Nelissen I., Rudd P. M., Dwek R. A., Opdenakker G. (2002). Biochemistry and molecular biology of gelatinase B or matrix metalloproteinase-9 (MMP-9). *Critical Reviews in Biochemistry and Molecular Biology*.

[25] Russell R. E. K., Culpitt S. V., DeMatos C. (2002). Release and activity of matrix metalloproteinase-9 and tissue inhibitor of metalloproteinase-1 by alveolar macrophages from patients with chronic obstructive pulmonary disease. *American Journal of Respiratory Cell and Molecular Biology*.

[26] Linder R., Rönmark E., Pourazar J., Behndig A., Blomberg A., Lindberg A. (2015). Serum metalloproteinase-9 is related to COPD severity and symptoms - cross-sectional data from a population based cohort-study. *Respiratory Research*.

[27] Abd El-Fatah M. F., Ghazy M. A., Mostafa M. S., El-Attar M. M., Osman A. (2015). Identification of MMP-9 as a biomarker for detecting progression of chronic obstructive pulmonary disease. *The International Journal of Biochemistry & Cell Biology*.

[28] Zhou X.-M., Hou G., Gu D.-X., Wang Q.-Y., Zhao L. (2017). Peroxisome proliferator-activated receptor-*γ* in induced sputum is correlated with MMP-9/TIMP-1 imbalance and formation of emphysema in COPD patients. *Journal of Thoracic Disease*.

[29] Li Y., Lu Y., Zhao Z. (2016). Relationships of MMP-9 and TIMP-1 proteins with chronic obstructive pulmonary disease risk: A systematic review and meta-analysis. *Journal of Research in Medical Sciences*.

[30] Montaño M., Sansores R. H., Becerril C. (2014). FEV1 inversely correlates with metalloproteinases 1, 7, 9 and CRP in COPD by biomass smoke exposure. *Respiratory Research*.

[31] Lo C., Huang H., He J. (2018). Increased matrix metalloproteinase-9 to tissue inhibitor of metalloproteinase-1 ratio in smokers with airway hyperresponsiveness and accelerated lung function decline. *International Journal of Chronic Obstructive Pulmonary Disease*.

[32] Kleniewska A., Walusiak-Skorupalh J., Piotrowski W., Nowakowska-Świrta E., Wiszniewska M. (2016). Comparison of biomarkers in serum and induced sputum of patients with occupational asthma and chronic obstructive pulmonary disease. *Journal of Occupational Health*.

[33] de Torres J. P., Casanova C., Pinto-Plata V. (2011). Gender differences in plasma biomarker levels in a cohort of COPD patients: A pilot study. *PLoS ONE*.

