# Supplementary material for: Impact of Matrix Metalloproteinase 9 on COPD Development in Polish Patients: Genetic Polymorphism, Protein Level, and Their Relationship with Lung Function
Source: Biomed Res Int. 2018 Dec 10;2018:6417415. doi: 10.1155/2018/6417415 (PMC6311264; doi:10.1155/2018/6417415)
Supplement: Supplementary Materials — Table 1S. Logistic regression analysis of association between 1562C/T SNP of MMP9 gene (rs3918242) and COPD, the multiple inheritance models. Figure 1S. Gender-related frequency of alleles of MMP9 rs3918242 polymorphism (-1562C/T) in patients with COPD and controls groups Figure 2S. Gender-related frequency of genotypes of MMP9 rs3918242 polymorphism (-1562C/T) in patients with COPD and controls groups. Figure 3S. Gender-related MMP-9 protein levels in patients with COPD and controls groups. Figure 4S. Gender-related MMP-9/TIMP1 complex levels in patients with COPD and controls groups. Figure 5S. Gender-related MMP-9/TIMP2 complex levels in patients with COPD and controls groups. [file 6417415.f1.docx]

Table 1S. Logistic regression analysis of association between -1562C/T SNP of *MMP9* gene (rs3918242) and COPD – the multiple inheritance models

| Model | rs3918242  genotypes | Frequency (Number of positive) | | COPD vs. CTR | | |
| --- | --- | --- | --- | --- | --- | --- |
|  |  | COPD  N=335 | CTR  N=309 |  |  |  |
|  |  |  |  | *P* | OR | 95%CI |
| Codominant | CC | 76.1 (255) | 73.1 (226) | 0.62 | 1.00 | - |
|  | CT | 21.8 (73) | 23.9 (74) |  | 1.14 | 0.79-1.66 |
|  | TT | 2.1 (7) | 2.9 (9) |  | 1.45 | 0.53-3.96 |
| Dominant | CC | 76.1 (255) | 73.1 (226) | 0.38 | 1.00 | - |
|  | CT+TT | 23.9 (80) | 26.9 (83) |  | 1.17 | 0.82-1.67 |
| Recessive | CC+CT | 97.9 (328) | 97.1 (300) | 0.50 | 1.00 | - |
|  | TT | 2.1 (7) | 2.9 (9) |  | 1.41 | 0.52-3.82 |
| Over-dominant | CC+TT | 78.2 (262) | 76. (235) | 0.51 | 1.00 | - |
|  | CT | 21.8 (73) | 23.9 (74) |  | 1.13 | 0.78-1.63 |
| Log-additive |  |  |  | 0.34 | 1.16 | 0.85-1.59 |

N, number of individuals; COPD, chronic obstructive pulmonary disease groups; CTR, control group

Figure 1S. Gender-related frequency of alleles of *MMP9* rs3918242 polymorphism (-1562C/T) in patients with COPD and controls groups

Legends: COPD, patients with chronic obstructive pulmonary disease; CTR, controls; CTR - sm, controls smokers; CTR – non-sm, controls non-smokers

Figure 2S. Gender-related frequency of genotypes of *MMP9* rs3918242 polymorphism (-1562C/T) in patients with COPD and controls groups.

Legends: COPD, patients with chronic obstructive pulmonary disease; CTR, controls; CTR - sm, controls smokers; CTR – non-sm, controls non-smokers

Figure 3S. Gender-related MMP-9 protein levels in patients with COPD and controls groups. Legends: men – COPD, men with chronic obstructive pulmonary disease; women - COPD, women with chronic obstructive pulmonary disease; men-ctr, controls men; women-ctr, controls women; men – sm, controls men – smokers; women – sm, controls women – smokers; men – non-sm, controls men – non-smokers; women – non-sm, controls women – non-smokers

Figure 4S. Gender-related MMP-9/TIMP1 complex levels in patients with COPD and controls groups.

Legends: men – COPD, men with chronic obstructive pulmonary disease; women - COPD, women with chronic obstructive pulmonary disease; men-ctr, controls men; women-ctr, controls women; men – sm, controls men – smokers; women – sm, controls women – smokers; men – non-sm, controls men – non-smokers; women – non-sm, controls women – non-smokers

Figure 5S. Gender-related MMP-9/TIMP2 complex levels in patients with COPD and controls groups.

Legends: men – COPD, men with chronic obstructive pulmonary disease; women - COPD, women with chronic obstructive pulmonary disease; men-ctr, controls men; women-ctr, controls women; men – sm, controls men – smokers; women – sm, controls women – smokers; men – non-sm, controls men – non-smokers; women – non-sm, controls women – non-smokers
